# Carbapenemase-producing Bacteria in Patients Hospitalized Abroad, France

**DOI:** 10.3201/eid2007.131638

**Published:** 2014-07

**Authors:** Fabrice Compain, Dominique Decré, Isabelle Frazier, Astrid Ramahefasolo, Marie Lavollay, Etienne Carbonnelle, Hidayeth Rostane, Arzu Tackin, Anne Berger-Carbonne, Isabelle Podglajen

**Affiliations:** Hôpital Européen Georges Pompidou, AP-HP, Paris, France (F. Compain, I. Frazier, A. Ramahefasolo, M. Lavollay, E. Carbonnelle, H. Rostane, A. Tackin, A. Berger-Carbonne, I. Podglajen);; Université Pierre et Marie Curie, Paris (D. Decré);; Université Paris Descartes, Paris (M. Lavollay, E. Carbonnelle, I. Podglajen);; Collège de France, Centre de Recherche Interdisciplinaire en Biologie, Paris (I. Podglajen);; Institut National de la Santé et de la Recherche Médicale, Paris (M. Lavollay, E. Carbonnelle, I. Podglajen)

**Keywords:** carbapenemase, colonization, glycopeptide resistance, hospitalization history, repatriated patients, France, bacteria, antimicrobial resistance

**To the Editor:** The emergence and rapid worldwide dissemination of carbapenemase-producing bacteria (CPB), especially carbapenemase-producing *Enterobacteriaceae* (CPE), have prompted public health authorities to reconsider prevention strategies to control the spread of these organisms (*1**–**5*). In France, national guidelines recommend systematic screening for commensal CPE and glycopeptide-resistant enterococci (GRE) in all patients admitted to hospitals who have been hospitalized in other countries during the preceding 12 months (*6**,**7*) (repatriated patients), independently of whether transfer was direct from hospital to hospital (DT) or not (NDT). These guidelines also recommend implementation of presumptive patient isolation and contact precautions on admission (*6**,**7*). We conducted a 33-month survey at Hôpital Européen Georges Pompidou (HEGP), a university teaching hospital in Paris, of CPE and GRE in repatriated patients; we also investigated incidence of extended-spectrum β-lactamase (ESBL)–producing *Enterobacteriaceae* and carbapenemase-producing *Acinetobacter baumannii* and *Pseudomonas* spp. in the same patient group.

During November 2010–July 2013, a total of 541 patients who had previously been hospitalized in a total of 71 other countries were admitted to HEGP. Rectal swab specimens were taken from 510 patients; 82 (16.1%) were DT, 415 (81.4%) were NDT, and 13 (2.5%) had an unclear history of transfer. Median patient age was 61 (range 12–98) years; 70% of patients were male. Results of screening by using antibiotic-containing Luria Bertani broths for enrichment and plating on selective media were negative for 354 (69.4%) of the 510 patients surveyed; 33 (6.5%; 16 DT, 17 NDT) patients were colonized with >1 CPB and/or GRE and 123 (24.1%; 22 DT, 99 NDT, 2 unclear) with ESBL producers only. More specifically, 19.5% (16/82) of DT patients and 4.1% (17/415) of NDT patients were colonized with CPB and/or GRE (p<10^−5^ by χ^2^ test); 26.8% (22/82) of DT patients and 23.9% (99/415) of NDT patients were colonized with ESBL producers only (p = 0.67). Characteristics of the 33 patients carrying CPB and/or GRE are shown in the Table. Of all isolates, 191 produced ESBLs only.

**Table:** Clinical and laboratory data on 33 patients hospitalized in France who were previously hospitalized in other countries and were carrying carbapenemase-producing bacteria, glycopeptide-resistant enterococci, or both*

Patient no.	Year of initial hospital admission	Patient transfer status	Country of hospitalization	Species of infection	β-lactamase content		Glycopeptide resistance gene
ESBL	Carbapenemase
1†	2010	DT	Egypt	*E. coli*	Pos	OXA-48	
2†	2010	DT	Thailand	*A. baumannii*	Pos	OXA-23	
3†	2010	DT	Iraq	*A. baumannii*	Neg	OXA-23	
				*E. faecium*			*vanA*‡
4	2010	NDT	USA	*E. faecium*			*vanA*
5	2011	NDT	Morocco	*K. pneumoniae*	Neg	OXA-48	
				*K. pneumoniae*	Neg	OXA-48	
6†	2011	DT	Senegal	*K. pneumoniae*	Pos	OXA-48	
7	2011	DT	Congo	*E. faecium*			*vanA*‡
8†	2011	NDT	Benin	*A. baumannii*	Neg	OXA-23‡	
9	2011	NDT	Kuwait	*P. aeruginosa*	Neg	VIM-2	
10†	2011	NDT	Kuwait	*K. pneumoniae*	Neg	OXA-48	
11†	2011	NDT	Kuwait	*P. aeruginosa*	Neg	VIM-2	
12†	2011	NDT	Kuwait	*E. faecium*			*vanA*
13†	2011	DT	Libya	*K. pneumoniae*	Pos	OXA-48	
14†	2011	DT	Libya	*E. coli*	Pos	OXA-48	
				*K. pneumoniae*	Pos	OXA-48	
				*A. baumannii*	Neg	OXA-23	
				*E. faecium*			*vanA*
15	2011	NDT	Saudi Arabia	*E. faecium*			*vanA*
16†	2011	NDT	Pakistan	*E. faecium*			*vanA*
17	2011	NDT	Italy	*A. baumannii*	Neg	OXA-23	
				*K. pneumoniae*	Neg	KPC-3	
18†	2011	DT	Spain	*K. pneumoniae*	Neg	OXA-48	
19	2011	NDT	Israel	*K. pneumoniae*	Neg	KPC-3	
20†	2012	DT	Egypt	*A. baumannii*	Neg	NDM-1	
				*A. baumannii*	Neg	NDM-1	
				*P. putida*	Neg	VIM-2	
21†	2012	DT	Tunisia	*E. coli*	Pos	OXA-48	
				*K. pneumoniae*	Pos	OXA-48	
				*K. pneumoniae*	Neg	OXA-48	
22†	2012	NDT	Tunisia	*A. baumannii*	Neg	OXA-23	
23†	2012	DT	India	*E. coli*	Neg	NDM-1	
				*M. morgannii*	Pos	NDM-1	
				*P. aeruginosa*	Neg	VIM-2	
24†	2012	NDT	Cambodia	*E. coli*	Neg	NDM-4‡	
				*E. coli*	Pos	OXA-48‡	
25†	2012	DT	Sri Lanka	*E. coli*	Pos	OXA-48‡	
				*K. pneumoniae*	Pos	OXA-181‡	
26	2013	NDT	Algeria	*E. coli*	Neg	OXA-48	
27†	2013	NDT	Algeria	*E. coli*	Neg	OXA-48	
28	2013	DT	Tunisia	*A. baumannii*	Neg	NDM-1	
29†	2013	DT	Libya	*K. pneumoniae*	Pos	OXA-48	
30	2013	DT	Libya	*K. pneumoniae*	Pos	OXA-48	
				*K. pneumoniae*	Pos	OXA-48	
31	2013	NDT	India	*E. coli*	Pos	OXA-181	
				*E. coli*	Neg	NDM-7‡	
				*K. pneumoniae*	Neg	NDM-7‡	
32	2013	NDT	Georgia	*P. aeruginosa*	Pos	VIM-2‡	
33†	2013	DT	Montenegro	*E. faecium*			*vanA*‡

*ESBL, extended-spectrum β-lactamase; DT, direct transfer from hospital abroad to HEGP; *E. coli, Echerichia coli*; Pos, positive; *A. baumannii, Acinetobacter baumannii*; Neg, negative; *E. faecium, Enterococcus faecium*; NDT, nondirect transfer (hospitalized abroad within 12 mo before transfer to HEGP); *K. pneumoniae, Klebsiella pneumoniae*; *P. aeruginosa; Pseudomonas aeruginosa*; *P. putida, Pseudomonas putida*; *M. morganii, Morganella morganii*. †Patient carrying an ESBL producer in addition to the carbapenemase-producing bacteria and/or glycopeptide-resistant enterococci. ‡Resistance gene not reported previously in the country of initial hospitalization.

Rates of resistance for ESBL*-*producing *Enterobacteriaceae* and CPE were, respectively, 53.1% and 57.1% to gentamicin, 16.7% and 32.1% to amikacin, 77.1% and 82.1% to nalidixic acid, 63% and 75% to levofloxacin, and 70.3% and 75% to ciprofloxacin. The *Pseudomonas* spp. and *A. baumannii* isolates were also multidrug resistant; all isolates were colistin susceptible.

Among the 33 colonized patients, 13 (39.4%) were not infected; 1 of the uninfected patients died. Seven patients were infected with >1 CPB (health care–related in 2 patients, 1 of whom died), 4 patients with ESBL-producing *Enterobacteriaceae* (health care–related in 1 patient, who died), and 9 patients with other bacteria (health care–related in 4 patients, 1 of whom died). No patients were infected with GRE. Overall, 60.6% of colonized patients were infected and 12.1% died; 35% (7/20) of the infections were health care–related (3 urinary tract device–related infections, 2 cases of ventilator-associated pneumonia, 1 infection at the site of a portacath, and 1 case of cellulitis).

Almost 25% of the repatriated patients carried ESBL-producing *Enterobacteriaceae* (mostly CTX-M-15 producers; Technical Appendix); 6.7% carried CPB and/or GRE. By comparison, during the study period, only 10.8% of 2,314 systematically screened patients in the medical and general surgery intensive care units at HEGP (repatriated patients excluded) carried ESBL-producing *Enterobacteriaceae*; 1 carried *vanA* *Enterococcus faecium* (data not shown). For patients with no record of hospitalization abroad, no CPE isolates were found; other bacterial isolates included 1 *vanA* *E. faecalis*, 13 *vanA E. faecium* (all known from previous outbreaks), 4 OXA-23–producing *A. baumannii*, and 4 VIM- and 1 IMP-producing *P. aeruginosa*.

Of the repatriated patients, 19.5% of DT patients (vs. 4.1% of NDT) and 23.9% (7 DT, 4 NDT) of those who were transferred to medical and general surgery intensive care units (ICUs) were CPB and/or GRE carriers. This finding highlights the role of severe underlying disease or injury and recent antimicrobial drug treatment. Among ICU patients, 3 died, most likely from underlying conditions, findings in line with the observation that carriage of or infection with multidrug-resistant bacteria is not the only predictor of death (*8*). Most of the 28 CPE isolates were resistant to fluoroquinolones and aminoglycosides except amikacin; 21 carried OXA-48–type genes, 7 of which were non-ESBL producers and were detected only around an ertapenem disk on Drigalski agar (Bio-Rad, Marnes-la-Coquette, France). All CPB, irrespective of species, showed imipenem hydrolysis in a recently described test (*9*) that was shortened and simplified by incubating colonies directly in antibiotic solution.

Although time-consuming and certainly perfectible, implementation of strict control measures to limit CPB and GRE spread (*6**,**7*) seems justified, a conclusion supported by the occurrence, since November 2010, of just 1 cross-transmission–linked CPB outbreak in an ICU at HEGP (after urgent intervention for cardiac arrest). Of particular concern is the high proportion of OXA-48–producing isolates in persons with no documented link to repatriation in France (*10*). This finding could be explained in part by the historical and demographic relationships between France and North Africa, where prevalence of OXA-48 is high, reflected in results from patients repatriated from that part of the continent.

Technical Appendix β-lactamase profiles of multidrug-resistant Gram-negative bacilli isolated in 2010 and 2011, France.
