# Supplementary material for: Carbapenemase-producing Bacteria in Patients Hospitalized Abroad, France
Source: Emerg Infect Dis. 2014 Jul;20(7):1246–8. doi: 10.3201/eid2007.131638 (PMC4073856; doi:10.3201/eid2007.131638)
Supplement: Technical Appendix — β-lactamase profiles of multidrug-resistant Gram-negative bacilli isolated in 2010 and 2011, France. [file 13-1638-Techapp-s1.pdf]

Article DOI: http://dx.doi.org/10.3201/eid2007.131638

Carbapenemase-Producing Bacteria in Patients Hospitalized Abroad, France

Technical Appendix

Technical Appendix Table. β-lactamase profiles of multidrug-resistant Gram-negative bacilli isolated in 2010 and 2011, France*

Region of origin	No. culture-positive patients/total no. patients	Species	No. isolates	β-lactamase ESBL	β-lactamase Carbapenemase	β-lactamase pCASE
Africa	33/87	*E. coli*	18	CTX-M-15		
			2	CTX-M-1		
			2	SHV-12		
			1	CTX-M-3	OXA-48	
			1	CTX-M-14		
			1	CTX-M-15 TEM-169		
			1	CTX-M-15		CMY-2
			1	CTX-M-24	OXA-48	
			1	ND		
		K. pneumoniae	12	CTX-M-15		
			2	CTX-M-15	OXA-48	
			2		OXA-48	
			1	CTX-M-15	OXA-48	CMY-2
			1	ND		
		E. cloacae	3	CTX-M-15		
			1	ND		
		M. morganii	2	CTX-M-15		
		A. baumannii	2		OXA-23	
Asia	26/60	*E. coli*	15	CTX-M-15		
			3	CTX-M-14		
			2	CTX-M-1		
			1	CTX-M-2		
			1	CTX-M-15		CMY-42
			1	ND		
		K. pneumoniae	6	CTX-M-15		
			1		KPC-3	
			1		OXA-48	
		P. mirabilis	1	CTX-M-55		
		A. baumannii	1	PER-7	OXA-23	
			1		OXA-23	
		P. aeruginosa	1	GES-1		
			2		VIM-2	
Europe	3/13	*E. coli*	1	CTX-M-27		
		K. pneumoniae	1	CTX-M-15		
			1		KPC-3	
			1		OXA-48	
		E. cloacae	1	CTX-M-15		
		A. baumannii	1		OXA-23	
North America	1/3	*E. cloacae*	1	CTX-M-15		
Oceania	1/2	*E. coli*	1	TEM-21		
		E. cloacae	1	TEM-21		

*ESBL, extended-spectrum β-lactamase; pCASE, plasmid-mediated cephalosporinase; ND, not determined.
